# Nutritional Aspects of Treatment in Epileptic Patients

**Published:** 2016

**Authors:** Danesh SOLTANI, Majid GHAFFAR POUR, Abbas TAFAKHORI, Payam SARRAF, Sama BITARAFAN

**Affiliations:** 1 Iranian Center of Neurological Research, Imam Khomeini Hospital, Tehran University of Medical Sciences, Tehran, Iran

**Keywords:** Epilepsy, Seizure, Nutritional status, Diet

## Abstract

Epilepsy is a neurological disorder characterized by interruption of normal neuronal functions that is manifested by behavioral disorders, changing of awareness level, and presence of some sensory, autonomic and motor symptoms or signs. It is resulted from many different causes. Many antiepileptic drugs (AEDs) are considered to manage epileptic attacks. Some of them change metabolism and absorption of many nutrients. Therefore, epileptic patients may be in higher risk of nutrient deficiency and its unwelcome effects. In the present paper, we intend to review the relationship between nutrition and epilepsy in two aspects. In one aspect we discuss the nutritional status in epileptic patients, the causes of nutritional deficiencies and the way of compensation of the nutrient deficiencies. It will guide these patients to have a healthy life. In another aspect we explain the role of some nutrients and specific diets in management of epileptic attacks. It can help to better control of epileptic attacks in these patients.

## Introduction

Epilepsy is a common and chronic neurological diseases (1), classified into several groups based on clinical characteristics (2-3). Etiology of epilepsy is not well known but genetic (4), physical and metabolic causes have been ascribed so far (5). Mutation in some genes encoded voltage-gated Na+ and k+ channel respectively plays an important role in molecular pathogenesis of some kinds of epilepsy (6-7). Physical causes such as trauma (8-9), stroke (10), infection (11-12) and tumors (9, 13) are also involved in the etiology of symptomatic epilepsy. Important metabolic causes result in reduction of oxygen supply in blood and (9) mitochondrial disorders which can leads to the lack of ATP needs for cellular metabolism (14-15). Aknown mechanism involved in pathogenesis of seizure related to nutritional status, is the imbalance between free radicals and antioxidant agents. In a study, level of zinc (Zn) decreased and level of copper (CU) increased in epileptic children before initiation of treatment with AEDs, however, serum level of iron was decreased in girls (16). Normal dietary intake of some minerals such as Zn, Cu and selenium (Se) needed in the normal function of antioxidative system, are essential for normal function of neurons and aid to treatment of seizure consecutively (17, 18). 

## Relationship between nutrition and epilepsy

**1. Nutritional status in epileptic patients**

**Antiepileptic drugs**

AEDsare classified in two categories. One is the liver “enzyme-inducing antiepileptic drugs” (EIAED) and another is the “non enzyme-inducing antiepileptic drugs”(NEIAED). Some of AEDs including phenytoin, phenobarbital, and carbamazepine that are in EIAED category induce catabolism of some nutrients. Some of AEDs are in NEIAED category including levetiracetam, valproate sodium, topiramate, clobazam, clonazepam, ethosuximide, gabapentin, lacosamide, lamotrigine, pregabalin, tiagabine vigabatrin and zonisamide have not serious effects on nutrients (19-22). In this article we review studies regarding the effects of AEDs on nutrient metabolism in epileptic patients.

## Antiepileptic drugs and nutrient deficiencies

**Vitamin deficiency**

Epileptic patients, treated with EIAEDs, are at risk for bone diseases like osteopenia, osteomalacia, rickets, and osteoporosis (23-27). The correlation between bone fractures and treatment with EIAEDs is reported (28-30). Bone fractures were correlated with the stored load of EIAEDs in these patients (31-32). The proposed mechanism is that EIAEDs may increase the function of the cytochrome p-450 enzymes which induce production of inactive form from the active form of vitamin D (vit D) (23, 33-35). In this way, absorption of the calcium (Ca) from gastrointestinal tract will be reduced. The reduction of the serum vit D and Ca absorption stimulate the release of parathyroid hormone (PTH) which results in higher uptake of Ca from bone (36-39). EIAEDs disturb Ca homeostasis and decrease serum level of Ca. This result is due to the effect of long-term therapy with anticonvulsant drugs on vitamin D metabolism (32, 36, 38, 40). Therefore patients on long-term treatment with EIAEDs should be followed up for serum vit D level and bone mineral density (BMD) (41-43). Ca and vit D supplementation may prevent vit D and Ca deficiencies and improve BMD in these patients (22, 44-45). Long term therapy with EIAEDs also decrease concentration of B vitamins including vitamin B1, B2, B6, B8 and B9 in epileptic patients and increase the aminothiol redox and induce hyper homocysteinemia consequently (20, 46-54). EIAEDs reduce serum biotin or vit B8 level and increase urinary excretion of its metabolites due to rise in biotin catabolism (55- 58). Deficiency of nicotinic acidor vit B3 is induced by valproate (59). Serum level of cobalamin or vit B12 was lower in patients treated with AEDs (52, 60-61). EIAEDs disturb the normal function of folateconjugase in intestine. Mentioned enzyme has key role in conversion of dietary folatepolyglutamates to folatemonoglutamate for better absorption. As a result, EIAEDs reduce folate absorption from folatepolyglutamates in foods (62). Among NEIAED, valproic acid inhibits glutamate formyltransferase enzyme and decrease the formation of active metabolite of folic acid that is named folinic acid (63). Epileptic women taken these drugs may be in high risk to give birth to the neural tube defect (NTD) infants because of low folate absorption (63). Low folic acid and vitamin B12 level include megaloblastic anemia with high mean corpuscular volume (MCV) and high plasma total homocysteine (Hcy) in these patients (25, 52, 60-61, 64-65). Folate and vitamin B12 deficiency may reduce the chromosomal stability, synthesis of myelin and synthesis of catecholamine, correlated with cognitive deficits and congenital malformations in addition to anemia and hyperhomocysteinemia (52). Studies recommend monitoring serum level of vit B9, vit B12 and serum Hcy. Supplementation with these vitamins improves the mentioned problems. This also can prevent epileptic patients from cardiovascular disease (52, 64, 66-68). EIAEDs increase catabolism of pyridoxine or vitB6 because of increasing activity of the oxidizing enzyme in the liver, inducing vitB6 deficiency and polyneuropathy consequently in patients with seizure. In addition, EIAEDs reduce the transsulfuration pathway which is effective in PLP synthesis (19-20). Deficiency of vitB6 decrease the seizure threshold (69-70) associated with higher Hcy concentrations (71). Pyridoxine supplementation may improve seizure threshold and hyperhomocysteinemia in these patients (20, 51). Supplementation with B-vitamins was recommended to patients on EIAEDs with hyperhomocysteinemia and high aminothiol redox (72). One of the important antioxidant agents known as a neuroprotective factors, is ascorbic acid or vit C. It collaborates with vitamin E for decrease oxidative stress, lipid peroxidation and strengthens of brain cell membranes (73). Furthermore, vit C is considered as an antiepileptic agent and a new treatment for seizure control due to induction of protective gene expression (74-75). Therefore, vit C supplementation may help to epileptic patients (76). EIAEDs with ability of lowering liver retinol (vit A) resources may be teratogenic (77). Usage of EIAEDs in patients with epilepsy may reduce liver vit A storage because of the movement of vit A from liver to the tissues or the stimulation of cytochrome p-450, reticulum endoplasmic enzymes, and increasing of serum retinolbinding protein (78). Therefore, EIAEDs and valproate, induce the liver enzymes that metabolize retinoic acids (RA) and lower the RA level in serum (77). As a result sufficient dietary intake of vit A is recommended to these patients. Neonates from pregnant epileptic mothers on anticonvulsant drugs are at higher risk of vitamin K deficiency however deficiency of vit K is not common in mothers (79). Vitamin K supplementation during pregnancy in epileptic mothers that are on EIAEDs will not lower the risk of vit K deficiency in neonates, but supplementation after birth in infants will be efficient (80).

## Mineral deficiency

Reports on the impact of antiepileptic drugs on the homeostasis of minerals are little and controversial (81-82). EIAEDs effect on Zn and Cu metabolism and induce Zn deficiency (75, 83). But in controversy Zn serum levels in these patients and healthy people are not significantly different (84-87). It was supposed that distribution of intracellular Zn was affected by AEDs (88). CU serum levels increase in epileptic patients because of increasing the ceruloplasmin synthesis and CU absorption (75, 83, 89). Patients with epilepsy are at risk of selenium (Se) and Zn deficiencies that have antioxidant function. Valproic acid, phenytoin, and carbamazepine produce higher reactive oxygen species (ROS) that use resources of Zn and Se but new epileptic drugs (e.g., topiramate and zonisamide) have not this effect. Se storage depletion may induce hepatotoxicity because of its antioxidant effects (53, 81-82). Carbamazepine monotherapy may maintain trace element and antioxidants resources rather than phenytoin (86). Phenytoin did not alter iron, magnesium (Mg) and Zn serum levels (75). However, high dietary intake of Zn or uncontrolled Zn supplementation can produce toxicity and induce some of central nervous system problem such as brain ischemia and epilepsy (90). Thus brain Zn homeostasis should be maintained for prevention and treatment of neurological disorders (91). Zn supplementation has no positive effects on BBB integrity and long term Zn supplementation has negative effect on Mg and Cu brain concentration in epileptic patients (16, 92). Generally, monitoring of dietary intake, serum level of nutrients and compensation of deficiencies is recommended in epileptic patients.

**1. Recommended Diets in epileptic patients**

Some patients are resistant to antiepilepticdrugs, then ketogenic diet can help to control their attacks (93). 

- Ketogenic diet

In patients with uncontrolled attacks, one of the most common and well-documented diets used as a treatment for drug-resistant epileptic patients is ketogenic diet (KD) (94-98). Ketogenic diet is consequential method to support of treatment in several types of epilepsy like atonic, mixed and myoclonic seizures (98-99). Ketogenic diet is supposed as a beneficial choice for treatment of patients with intractable seizures, instead of neurosurgery option, because of less adverse effects (95). Decrease in glucose level and ketosis are significant changes occurred during KD therapy. Lowering the serum level of glucose is more contributing to the control of seizures (100). The main mechanism of action in KD is not well known, but the high fat, low carbohydrate and enough protein content of the diet, lead to rise in plasma ketone bodies which play a helpful role in lowering the excitability of neurons and modifying seizure threshold. Moreover, ketone bodies can alter the amount of fluid, electrolytes and lipids intake on the way to help control of seizure attacks (95-97, 101-103). Ketogenic diet produces some mediators named acetoacetate and β-hydroxybutyrate (BHB), or both. These metabolites substitute for glucose as the substrate for energy producer, like the mechanism seen in long term hunger (104-106). Ketogenic diet is administered in two forms. One is the classic KD, includes longchain triglycerides (LCT) and second is medium-chain triglyceride (MCT) KD that contains fatty acids with 6–12 carbons. MCTs are included more common fatty acids, caprylic acid (CA8: 50–75% content), capric acid (CA10: 23–45%), caproic acid (CA6: 1–3%) and lauric acid (CA12: 1–5%) (94, 107-110). Co-administeration of CA8 and CA10, promote the anti-seizure effect of MCT ketogenic diet (111). Medium-chain triglycerides are more helpful in energy production because of faster production of ketone bodies and well-absorbed from GI in comparison to LCT in classic KD (99, 107-110, 112-116). The classic KD, with 3/1 ratio of fat/carbohydrate and MCT ketogenic diet have identical effect on control of seizure, but those have different effects on plasma lipid levels. Plasma cholesterol level in MCT regimen remains normal against the rise of that in the classic KD. In addition the amounts of free fatty acids in plasma increase on the MCT regimen lesser than classic KD (104). Medium-chain triglycerides are more soluble in aqueous media in comparison with LCTs. They are in free form in circulation and have high affinity to carriers which facilitate the transport from blood–brain barrier (114, 117-122). CA10 is agonist of PPARs which leads to rise of the metabolic enzymes in mitochondria of the neuronal cell (123). CA8 and CA10 increase the phosphorylation of p38 mitogen-activated kinase (MAPK) and extracellular signal regulated kinase (ERK) that act as anti-convulsant by altering the seizure inducer molecules (124-126). Another compound like branched medium chain fatty acids are new options to control epilepsy in some cases whom medium-chain triglyceride KD (MCTKD) is not sustainable (112). Octanoic acid or caprylic acid is one of the branched medium chain fatty acids achieved from the hydrolysesof coconut oil (110). Dravet syndrome (DS) is an infantile onset epileptic encephalopathy which is resistant to some antiepileptic drugs (127-128). One study compared KD with some AEDs used for DS patients. The efficacy of them was the same but the KD has lesser side effects (129). Fatty acids in KDs are saturated or monounsaturated so may have some complications (130). Polyunsaturated fatty acids (PUFAs) introduced as another option for aid to treat epilepsy. They includes omega-3 with the combination of docosahexanoic acid (DHA) and eicosapentaenoic acid (EPA) that are in seals and marine fishes, alphalinoleic acid (ALA) and in flaxseed, almonds, walnuts, as well as omega-6 which composed of linoleic acid (LA) and arachidonic acid (AA). Daily intake of 1 capsule includes of 1080 mg eicosapentaenoic acid and docosahexaenoic acid (low dose fish oil) is more effective to improve seizures than high dose (131-133). Classic KD is based on butter, cream, and olive oil (130). KD increase energy and GABA production due to changing in tricarboxcylic acid cycle. It also decreases production of ROS in brain. Ketogenic diet increases the expression of neuronal uncoupling proteins (UCPs) and some energy metabolism genes in mitochondria (134- 135). Ketogenic diet may produce diet limitations and deficiencies of some vitamins and minerals corrected with administration of vitamin and mineral supplements. Supplementation with vit B, vit D, Ca, Sel, Mg, Zn and phosphorus has been recommended in KD. In this way, one new carbohydrate-free multivitamin and mineral named NanoVM (Solace Nutrition, Rockville, MD, U.S.A.) have been designed for the KD in child. However, the appropriate multivitamin and mineral for epileptic patients on KD have already not been studied and designed (136-138). Some adverse effects of KD are growth (139), metabolic, gastrointestinal and urinary problems including hypercholesterolemia, hypocalcemia, hyperlipidemia (140-141), Secondary hypocarnitinemia (142), hypomagnesemia, lowered amino acid levels, acidosis (143-145), vomiting, constipation, diarrhea, and abdominal pain (145), kidney stone (136). Thus epileptic patients on KD should be observed by neurologist and dietitian for control of complications and nutritional deficiencies (136, 146). Few studies have been done on anticonvulsant complications of KD. In one study with a large population, half of patients improved over the 2 yr therapy, although the observations have some differences with those in children. Some complications of KD were thechanges of serum level HDL, triglycerideand carnitine in children so carnitine supplementation was needed. Supplementation with carnitine induce transportation of raised free fatty acids into mitochondria and decrease serum triglyceride (147). Ketosis alters the electrolyte, fluid and lipid concentration balance (95).

## Modified Atkins Diet and Low Glycemic Index Diet

Because of KD complications mentioned above, other types of diets were recommended in management of adolescents, adults (138, 153) and epileptic children along with AED (154). These diets were named Modified Atkins Diet (MAD) (103, 152) and Low Glycemic Index Treatment (LGIT). MAD is a modification of KD which includes PUFA (n-3 and n-6) groups that their protective role against seizure without any significant side effects has been demonstrated. This diet has been prepared of canola oil and diverse menu items such as fish and nuts (103, 130, 147). The mechanism of action in PUFAs-enriched diets is upregulation of some genes involved in mitochondrial metabolisms and stabilizing of neuron synapses which result in seizure hold up (103). PUFAs-enriched diet induces the production of mitochondrial uncoupling proteins (148). The agonistic function of ALA on PPARs is another mechanism which prevents seizure attacks by increasing the seizure thresholds (149). MAD is similar to classic KD. In MAD 10 g/d carbohydrate at the start of diet is raised to 20 g/d within 3 months although total daily intake of proteins, calories, and fluids were not decreased (155). Within LGIT, 40–60 g/d carbohydrate has been recommended. Carbohydrates with low glycemic indices are ones that increase blood glucose very low. Thus, blood glucose in patients on this diet is stable (152). Low Glycemic Index Treatment and Modified Atkins diet have well-controlled complications and lesser dietary restrictions in adult and children than KD (156-159). In conclusion, some AEDs can induce nutritional deficiencies. Then both nutritional status and serum levels of nutrients should be monitored in epileptic patients periodically deficiencies must be compensated with precise supplements. We recommend to supplementation with appropriate amounts of vitamins and minerals compound (multivitamin & mineral) included of vitamin A, D, E, C, B complex, Ca, Sel and Zn. Three alternatives of diets are considered for management of attacks in epileptic patients. These diets are KD, MAD and LGIT. We assessed these diets and recommended KD only in patients with no response to AEDs. But MAD and LGIT are appropriate in other patients on AEDs because of lower side effects and aid to treatment.
